# Risk of Functional Disorders and/or Thyroid Autoimmunity and Its Association with 25OH Vitamin D and Magnesium Levels: A Population-Based Case-Control Study

**DOI:** 10.3390/medsci14010143

**Published:** 2026-03-18

**Authors:** Hernando Vargas-Uricoechea, Alejandro Castellanos-Pinedo, Karen Urrego-Noguera, María V. Pinzón-Fernández, Ivonne A. Meza-Cabrera, Hernando Vargas-Sierra, Valentina Agredo-Delgado

**Affiliations:** 1 Metabolic Diseases Study Group, Department of Internal Medicine, Universidad del Cauca, Carrera 6 Nº 13N-50, Popayán 190001, Colombia; karenurrego@unicauca.edu.co (K.U.-N.); imeza@unicauca.edu.co (I.A.M.-C.); hdvargas@unicauca.edu.co (H.V.-S.); agredo.delgado.valentina@gmail.com (V.A.-D.); 2 Faculty of Medicine, Universidad del Sinú, Hospital San Jerónimo, Montería 230001, Colombia; acaspinedo@yahoo.es; 3 Health Research Group, Department of Internal Medicine, Universidad del Cauca, Popayán 190003, Colombia; mpinzon@unicauca.edu.co

**Keywords:** vitamin D, magnesium, thyroid, autoimmunity, thyroid function

## Abstract

Background/Objectives: Vitamin D (Vit-D) and magnesium (Mg) levels have been associated with an increased risk of developing functional thyroid disorders or autoimmune thyroid diseases (AITD). In this study, our objective was to evaluate if 25-hydroxyvitamin D (25OH Vit-D) and/or Mg levels are associated with an increased risk of functional thyroid disorders and/or AITD. Methods: A population-based case-control study was conducted, with a total of 1028 participants (514 cases and 514 controls). Blood concentrations of 25OH Vit-D, Mg, TSH, FT4, FT3, and thyroid autoantibodies (TPOAb, TgAb, and TRAb) were determined in the study participants. Results: Among the cases (in women), the prevalence of goiter, hypothyroidism, and thyroid autoantibody positivity was significantly higher. No differences were found in the prevalence of functional thyroid disorders or in thyroid antibody positivity (among cases) according to sex or age. The prevalence of thyroid antibody positivity (specifically TPOAb and/or TgAb) was significantly higher in cases with 25OH Vit-D and/or Mg deficiency. The 25OH Vit-D level that best discriminated the highest frequency of AITD was 23.5 ng/mL [AUC: 0.665 (95% CI: 0.636–0.694, *p* < 0.001)]; while for Mg it was 1.8 mg/dL [AUC: 0.697 (95% CI: 0.668–0.725, *p* < 0.001)], indicating that the model has weak discrimination (although better than chance), with good sensitivity and low specificity, being able to identify the vast majority of positive cases (with AITDs), at the cost of including a significant proportion of false positives. Conclusions: Overall, we found that low serum levels of 25OH Vit-D and/or Mg appear to be associated with a significantly increased risk of goiter, functional thyroid disorders (specifically hypothyroidism), and with greater positivity of thyroid antibodies.

## 1. Introduction

Autoimmune thyroid diseases (AITDs) are a group of organ-specific autoimmune diseases (AIDs), that involve a loss of self-tolerance towards thyroid antigens, mainly thyroglobulin (Tg), thyroid peroxidase (TPO), and the thyrotropin (TSH) receptor (TR) [1,2,3].

The prevalence of AITDs varies according to variables such as the geographical area studied, age, and sex, inter alia, and it has been described that they can affect 5% of the population, with marked differences according to sex, as they are more frequent in women than in men (5–15% vs. 1–5%, respectively) [4,5,6].

AITDs have various phenotypic manifestations or expressions, e.g., ranging from the isolated positivity of autoantibodies (Abs) directed against thyroid antigens (TgAb, TPOAb, and TRAb) to the presence of subclinical or primary thyroid dysfunction in hypothyroidism (in Hashimoto’s thyroiditis—HT) or hyperthyroidism (in Graves-Basedow disease—GBD) [6,7,8].

Despite their protean manifestations, AITDs have similar immunogenetic substrates and pathophysiological mechanisms, converging in terms of their genetic, epigenetic, environmental, and other aspects [9,10].

Among the environmental (nutritional) mechanisms, the population statuses of several micronutrients stand out [especially iodine, selenium, iron, zinc, magnesium (Mg), copper, vitamin A, vitamin B12, and vitamin D (Vit-D), inter alia], each of them has the ability to influence the synthesis, secretion and regulation of thyroxine (T4) and triiodothyronine (T3), as well as the immune responses to the thyroid gland [11,12,13].

Thus, excess or deficiency of these micronutrients has been linked to a higher frequency of AITDs and functional, morphological, and/or malignant thyroid alterations [12,13,14].

Based on the above information, Mg has been described as acting as a cofactor for several enzymes and enzymatic reactions, and also involved in the metabolism of T4 and T3; furthermore, the findings of some in vitro studies suggest that free Mg ions at the intracellular level act as second messengers in T lymphocyte (TL) and B lymphocyte (BL) activation, and are also associated with greater oxidative stress and an amplified inflammatory response [15,16].

Moreover, TL, BL, and antigen-presenting cells (APCs) express the enzyme 1α-hydroxylase; therefore, they are able to generate the active compound of Vit-D. The above information, in addition to the Vit-D receptor (VDR) also being expressed in these cells, suggests local effects on the immune system (immunomodulatory effects); accordingly, Vit-D acts as a modulator of the innate and adaptive immune system [15,16,17,18].

The authors of some studies have evaluated the association between a population’s Mg and/or Vit-D status and the risk of AITDs. Several of these have documented a possible relationship between deficiency in one or both micronutrients and a higher frequency of AITDs; however, the authors of other studies have not demonstrated such an association [11,12,13,16].

We hypothesize that the Mg and/or Vit-D population status is associated with alterations in thyroid function and an increased risk of AITDs. Therefore, in this study, we aim to determine whether serum Mg and/or 25-hydroxyvitamin D (25OHVit-D) concentrations are associated with alterations in thyroid function and/or the presence of AITDs.

## 2. Materials and Methods

### 2.1. Study Population

To evaluate the distribution and behavior of thyroid functional disorders and AITDs (and their potential associated factors) in southwestern Colombia, we conducted a population-based study between June 2018 and July 2025.

This study was carried out in accordance with the principles of the Declaration of Helsinki, with prior approval and authorization from the Research Ethics Committee (ID: 4656, 15 January 2018) of the University of Cauca, Colombia.

Adults (not institutionalized) residing in urban and rural areas (urban predominance) of the study region were included. Participants were proactively invited through conducting direct visits to different neighborhoods and communities, ensuring representative coverage.

Initially, 18,528 adults provided informed consent and completed a baseline health history questionnaire, of which 7319 individuals were excluded based on predefined eligibility criteria, yielding an initial eligible cohort of 11,209 potential participants.

**Figure 1:**
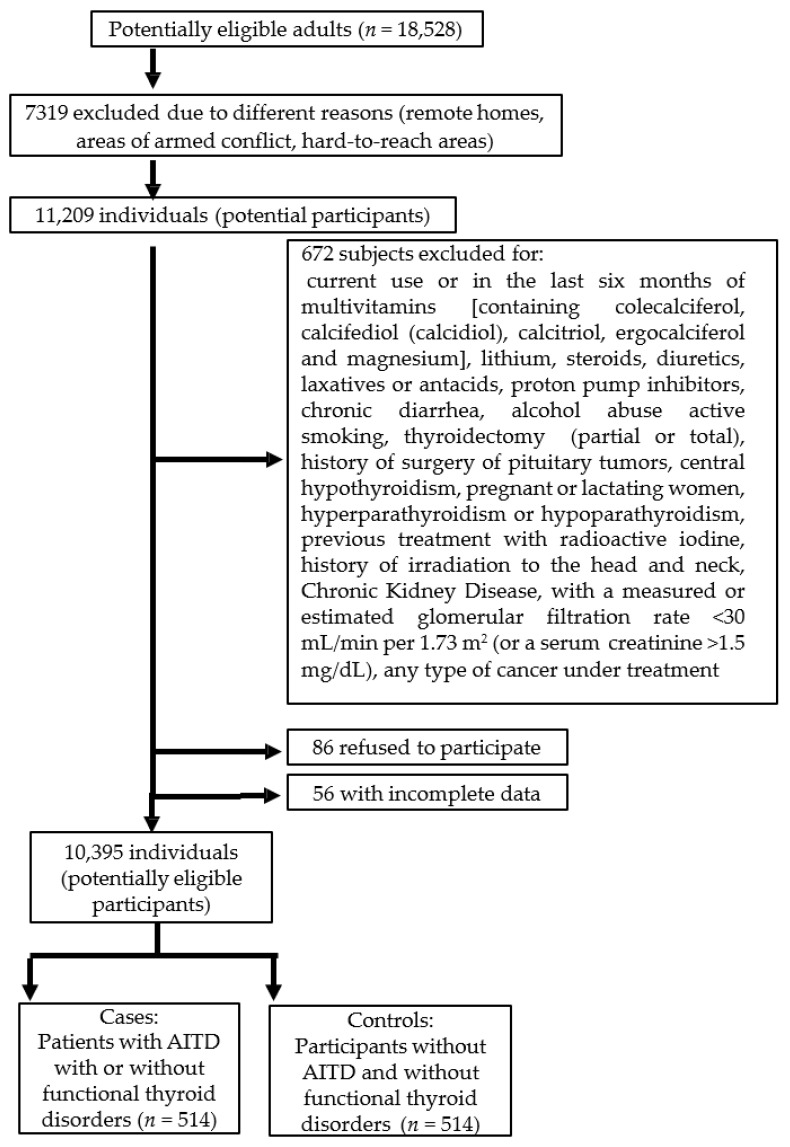
Flowchart and eligibility criteria for study participants.

Subsequently, 672 individuals were excluded (for various reasons), 86 declined to participate, and incomplete data were obtained from 56, leaving a final pool of 10,395 potentially eligible participants, from whom cases (*n* = 514) and controls (*n* = 514) were selected (Figure 1). These 1028 participants were those who met all the inclusion criteria and who had absolutely every one of the variables of interest to be evaluated (anthropometric, sociodemographic and laboratory).

The case-to-control ratio was 1:1, and they were matched according to the following potential confounding variables: age, sex, socioeconomic status (SES), origin (urban or rural), and body mass index (BMI).

### 2.2. Study Design

This research has an observational, analytical design, being a population-based case-control study.

### 2.3. Definition of AITDs and/or Thyroid Function (Normal or Abnormal)

We defined the presence of AITD according to whether there was positivity of at least one of the three thyroid Abs evaluated (TPOAb, TgAb and TRAb).

In order to evaluate and classify thyroid function (normal or abnormal), serum concentrations of TSH and free T4 (FT4) were determined, and the following definitions were established: euthyroidism (normal TSH and FT4); subclinical hypothyroidism (elevated TSH with normal FT4); primary hypothyroidism (elevated TSH with low FT4); primary hyperthyroidism (low TSH with elevated FT4); subclinical hyperthyroidism (low TSH with normal FT4), in the latter case, the serum concentration of free T3 (FT3) was also determined, in order to rule out (or confirm) the presence of T3-related hyperthyroidism [19,20,21].

### 2.4. Exclusion Criteria

To select both study groups, individuals who had been exposed to medications or health conditions that could potentially affect thyroid function in the last three months were excluded (Figure 1).

These exclusion factors were identified through self-reported information from the patient.

### 2.5. Sociodemographic and Anthropometric Data

Both cases and controls underwent a structured interview to assess sociodemographic data: age, height, weight, BMI (in kg/m^2^), and blood pressure (BP). Neck inspection and palpation were also performed to determine the presence of goiter (according to World Health Organization criteria) [22].

### 2.6. Laboratory Methods

All blood samples were obtained by venipuncture after fasting (7:00–9:00 a.m.), then centrifuged, frozen (at −80 °C), and stored. The measurement of variables such as TPOAb, TgAb, TRAb, TSH, FT4, and FT3 was performed using a chemiluminescent immunoassay (IMMULITE^®^ 2000 Systems Analyzers; Siemens, Munich, Germany).

Positivity for TPOAb, TgAb, and TRAb was established when the values obtained were ≥8.0 IU/mL, ≥18 IU/mL, and >1.75 IU/mL, respectively [23].

Meanwhile, the concentrations of TSH, FT4, and FT3 (considered normal) were: 0.4–4.5 mIU/L; 0.89–1.76 ng/dL; and 3.5–8.3 pmol/L, respectively, with their coefficients of variation (4.96%, 8.0% and 5.3%, respectively), while for TSH, FT4, and FT3 they were 4.6%, 6.4%, and 6.7%, respectively (manufacturer’s cutoff values) [23].

Serum Mg concentration was determined by spectrophotometry (normal reference range: 1.7–2.4 mg/dL). The coefficient of variation was 2.1%, with a detection limit of 0.1 mg/dL and a linearity limit of 4 mg/dL. Mg in the sample reacts with Xylidyl blue in an alkaline medium. This creates a colored complex that is measured by spectrophotometry (typically around 500–660 nm). The reagent formulation includes chelating agents like EGTA to remove interference from calcium, ensuring the color change is proportional to the Mg concentration [24].

To evaluate Vit-D status, the serum concentration of 25OH Vit-D was determined by means of a competitive chemiluminescence immunoassay.

The 25OH Vit-D assay is a two-incubation chemiluminescence immunoassay for the quantitative determination of total 25OH Vit-D in human serum. In the first incubation, the 25OH Vit-D is dissociated from its binding protein by the displacing reagent, and binds to the 25OH Vit-D antibody on the magnetic microbeads forming an antibody–antigen complex. Following a second incubation, the 25OH Vit-D labeled ABEI are added. The rest of the unbound material is removed during a wash cycle.

Subsequently, the Starter 1+2 is added to initiate a flash chemiluminescent reaction. The resulting chemiluminescent reaction is measured as relative light units (RLUs), which is inversely proportional to the concentration of 25OH Vit-D present in the sample (or calibrator/control, if applicable). The coefficient of variation was 6.28%, and the normal range was defined as 20–50 ng/mL [24,25]. The manufacturers for the laboratory analyzers of Mg and 25OH Vit-D were BioSystems and BioMérieux, respectively.

Taking into account that Colombia experiences a relatively uniform climate throughout the year (due to its equatorial location) and does not have distinct seasons like some other countries. However, the climate is divided into dry seasons from December to January and July to August, and rainy seasons from April to May and October to November.

In this study, all blood samples were collected continuously throughout the twelve months of the year (during the entire study period).

### 2.7. Statistical Analysis

In this study, data were collected from 1028 participants (using Windows Excel, Microsoft 2020) and processed using SPSS version 25.0 software (IBM-SPSS Inc., Chicago, IL, USA). All collected data (100%) underwent quality control, which included creating a descriptive analysis of each variable to cleanse and correct data; identify missing values, omissions, and blank cells; and determine which records met previously established operational definitions.

To describe the sociodemographic and clinical characteristics of the study population, descriptive statistics were used for numerical (range, mean ± standard deviation, quartiles) and categorical indicator variables (prevalence and percentage distributions). The 95% confidence intervals (CI) were calculated for the prevalence values obtained.

To perform the analysis, evaluating the association between dichotomous categorical variables, parametric and nonparametric statistical significance tests were used, depending on whether the statistical assumptions for their application were met (Chi-square and Fisher’s exact tests). To measure the strength of the association, the odds ratio (OR) and its 95% confidence interval (CI) were used.

The Shapiro–Wilk test was used, with the null hypothesis that the sample came from a normal distribution, with an alternative hypothesis that the distribution was non-normal. For the numerical variables, a bivariate analysis was performed (based on Student’s *t*-test, one-way analysis of variance, and linear regression). A multivariate binary logistic regression model was used to determine which characteristics were significantly associated with functional alterations and thyroid autoimmunity.

Variables that were significantly associated in the bivariate analysis (*p*-value < 0.05) were described and introduced. Using clinical measurements of Mg and 25OH Vit-D, receiver operating characteristic (ROC) curves were obtained to analyze the cutoff points, their sensitivity and specificity, and the area under the curve (AUC) to assess their predictive value.

A priori, a significance level of α = 0.05 was determined; therefore, the differences found between the participants (cases and controls) were evaluated and analyzed based on the magnitude of the effect.

## 3. Results

### 3.1. Anthropometric and Sociodemographic Characteristics, Prevalence of Functional Thyroid Disorders and Positivity of Thyroid Abs

Among participants, the mean age was 47.95 years and there was a higher proportion of women (most belonged to the low-SES group and lived in urban areas). The mean BMI was 27.7 kg/m^2^ and the mean BP was 128.8/73.2 mmHg (Table 1).

### 3.2. Prevalence of Goiter According to Age and Sex

Compared to the controls, the prevalence of goiter was significantly higher in the cases [64.6% vs. 11.5%; OR: 14.0; 95% CI: 10.2–19.6]; in patients < 50 years of age, it was 69.8% in cases and 9.5% in controls (*p* < 0.001); in women, the prevalence was 75.6% (in cases) and 9.4% (in controls) (*p* < 0.001); whereas in men, it was 60% and 9.5% (in cases and controls, respectively) (*p* < 0.001) (Table 2). In patients aged ≥ 50 years, the prevalence of goiter was 58.1% in cases and 14% in controls (*p* < 0.001); in women, it was 57.1% and 10.9% (*p* < 0.001); while in men, it was 49.8% and 16.8% (*p* < 0.001). The ORs of goiter prevalence in women (vs. men) were 3.5 (95% CI: 2.9–4.3; *p* < 0.001) for those <50 years, and 2.5 (95% CI: 2.0–2.9; *p* < 0.001) for those >50 years, respectively.

### 3.3. Prevalence of Thyroid Functional Disorders and Thyroid Abs Positivity

A significantly higher prevalence of subclinical hypothyroidism, primary hypothyroidism, and goiter was also documented in women (among cases). No differences were found in the prevalence of other thyroid disorders or in thyroid Abs positivity (among cases) based on sex or age (<50 vs. ≥50 years) (Table 2).

### 3.4. Prevalence of Normal (Or Deficient) 25OH Vit-D and Mg Levels According to Age

The prevalence of a normal 25OH Vit-D level (20–50 ng/mL) was 62.5% (in cases) vs. 93.6% (in controls), while the prevalence of 25OH Vit-D deficiency (<20 ng/mL and <12 ng/mL) was 37.5% vs. 6.4% and 5.1% vs. 0.6% in cases and controls, respectively.

The prevalence of 25OH Vit-D deficiency (<20 ng/mL) in participants aged < 50 years was 36.5% and 5.3% (in cases and controls, respectively) [OR: 10.3 (95% CI: 5.8–18.3; *p* < 0.001)], and in participants aged ≥ 50 years it was 38.9% (in cases) and 7.9% (in controls) [OR: 7.4 (95% CI: 4.3–12.9; *p* < 0.001)].

Among cases, the OR of having a 25OH Vit-D level < 20 ng/mL was 8.8 (95% CI: 5.9–13.0), while that of a level < 12 ng/mL (compared to a level of 12–50 ng/mL) was 9.1 (95% CI: 5.7–30.2; *p* < 0.001).

The prevalence of Mg deficiency (<1.7 mg/dL) in the total population was 13% (95% CI: 10.9–15.1), while that of normal Mg levels (1.7–2.4 mg/dL) was 78.9% (in cases) and 94.9% (in controls). However, the deficiency prevalence was 21.1% (in cases) vs. 5.1% (in controls); OR: 5.0; 95% CI: 3.2–7.8; *p* < 0.001 (in the total population); OR: 6.4; 95% CI: 3.0–13.2; *p* < 0.001 (in participants aged < 50 years), and OR: 4.3; 95% CI: 2.4–7.7; *p* < 0.001 (in participants aged ≥ 50 years).

The combined prevalence of Mg deficiency (<1.7 mg/dL) and 25OH Vit-D deficiency (<20 ng/dL) was 9.9% (in the total population); while it was 18.5% (in cases) and 1.4% (in controls); OR: 4.9; 95% CI: 2.7–7.6; *p* < 0.001 (Table 3).

### 3.5. Prevalence of Thyroid Abs Positivity According to 25OH Vit-D and Mg Levels (In Cases)

In cases, the prevalence of thyroid Abs positivity was significantly higher in participants with 25OH Vit-D levels from ≥12 to <20 ng/mL and in those < 12 ng/mL (compared to 20–50 ng/mL) in relation to TPOAb (but not for TgAb or TRAb).

The findings were similar in participants with Mg deficiency (compared to those with normal levels). Meanwhile, in participants with combined Mg and 25OH Vit-D, the prevalence of TPOAb and TgAb positivity was significantly higher (Table 4).

### 3.6. 25OH Vit-D and Mg Levels and Risk of Thyroid Dysfunction

Among the cases with 25OH Vit-D levels < 20 ng/mL, the OR of presenting with hypothyroidism (subclinical or primary) was 3.98 (95% CI: 2.87–4.82; *p* < 0.001); for those with Mg levels < 1.7 mg/dL, it was 3.2 (95% CI: 1.96–5.41; *p* < 0.001) for subclinical or primary hypothyroidism; and in those with combined Mg and 25OH Vit-D deficiency (<20 ng/mL), it was 3.81 (95% CI: 2.81–4.9; *p* < 0.001) for subclinical or primary hypothyroidism.

Among the cases with 25OH Vit-D levels < 20 ng/mL or with Mg levels < 1.7 mg/dL, the ORs of presenting with hyperthyroidism (subclinical or primary) were 1.06 (95% CI: 0.87–2.02; *p* = 0.32) and 1.1 (95% CI: 0.69–2.08; *p* = 0.28), respectively.

Meanwhile, the OR for participants with combined Mg and 25OH Vit-D deficiency (<20 ng/mL) was 1.31 (95% CI: 0.91–2.46; *p* = 0.09).

### 3.7. 25OH Vit-D and Mg Levels and AITD Risk

A complementary analysis was developed through an ROC curve analysis, which evaluated different cutoff points in the levels of 25OH Vit-D and Mg (AUC), with the purpose of determining which one best determined the highest frequency of AITDs.

**Figure 2:**
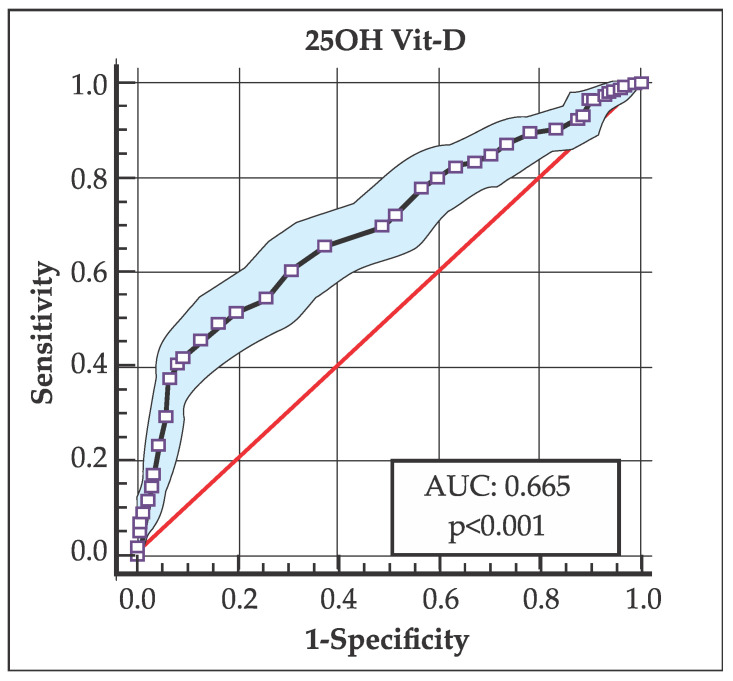
Area under the curve (AUC), showing that the cutoff point for the 25OH Vit-D level that best discriminates the highest frequency of AITD is 23.5 ng/mL [AUC: 0.665 (CI 95%: 0.636–0.694; *p* < 0.001)].

In this sense, the 25OH Vit-D level that best discriminated the highest frequency of AITDs was 23.5 ng/mL [AUC: 0.665 (95% CI: 0.636–0.694; *p* < 0.001); sensitivity: 0.928, specificity: 0.300 (Youden index: 0.228)] (Figure 2).

**Figure 3:**
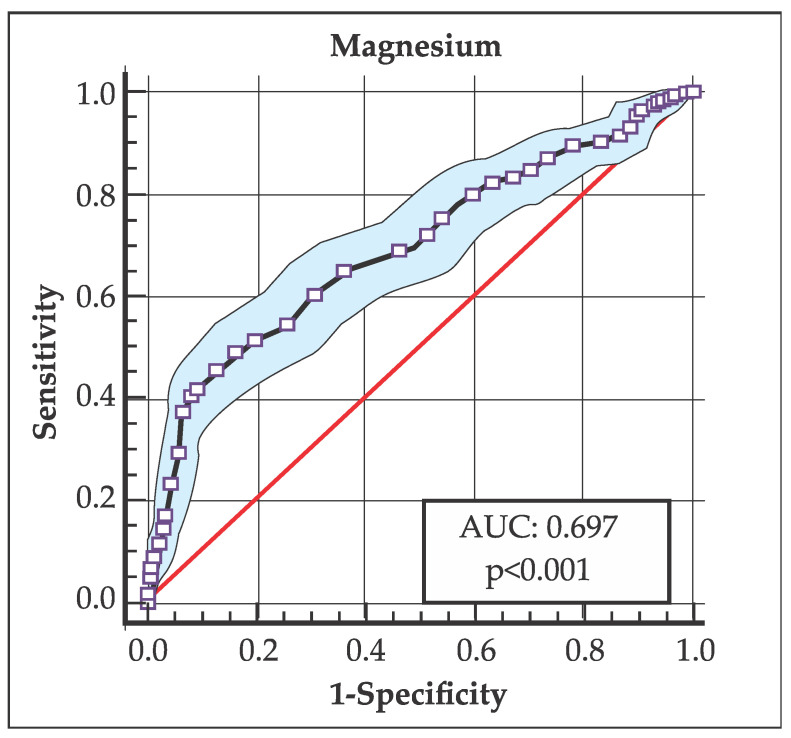
Area under the curve (AUC), showing that the cutoff point for the Mg level that best discriminates the highest frequency of AITD was 1.8 mg/dL [AUC: 0.697 (CI 95%: 0.668–0.725; *p* < 0.001).

Meanwhile, the Mg level that best discriminated the highest frequency of AITDs was 1.8 mg/dL [AUC: 0.697 (95% CI: 0.668–0.725; *p* < 0.001); sensitivity: 0.874, specificity: 0.457 (Youden index: 0.332)] (Figure 3).

When performing the logistic regression analysis (multivariate), we found that the risk of presenting with an AITD was significantly higher in cases with 25OH Vit-D levels <23.5 ng/mL [OR: 8.7 (95% CI: 5.9–13.0; *p* < 0.001)], with Mg levels < 1.8 mg/dL [OR: 5.0 (95% CI: 3.2–7.8; *p* < 0.001)], and with combined Mg (<1.7 mg/dL) and 25OH Vit-D deficiency (<20 ng/mL) [OR: 6.4 (95% CI: 4.1–7.7; *p* < 0.001)].

## 4. Discussion

Previous studies have documented conflicting results regarding low serum levels of 25OH Vit-D and an increased risk of AITD; most of the studies with negative results were observational with small sample sizes (and involved participants with other AIDs), while in studies with larger sample sizes (and in intervention studies with Vit-D supplementation), an association was found between Vit-D deficiency and a higher risk of AITDs.

In general, clinical trials involving Vit-D supplementation describe a significant reduction in TPOAb and TgAb positivity, along with a reduction in TSH levels and an increase in FT4 and FT3 levels (in patients with established hypothyroidism undergoing levothyroxine replacement therapy) [26,27,28,29,30,31].

In our study, we found a high prevalence of 25OH Vit-D deficiency, which was significantly higher in cases (and independent of age), documenting a significant association between 25OH Vit-D deficiency (<20 ng/mL) and higher prevalence of hypothyroidism (subclinical or primary), as well as higher thyroid Abs positivity (specifically TPOAb); additionally, we found that a cutoff point of <23.5 ng/mL (25OH Vit-D value) best discriminates the risk of presenting with an AITD.

Moreover, the higher prevalence of goiter and hypothyroidism in women has been documented in other studies. However, population-based studies have also been conducted in Colombia demonstrating a high rate of goiter, and it has been suggested that thyroid autoimmunity, the presence of possible goitrogens, and endocrine disruptors are the cause (linked to a higher frequency of deficiencies in other micronutrients) [23,32,33,34,35].

In this regard, several systematic reviews and meta-analyses have been carried out evaluating the possible associations between Vit-D deficiency and AITDs and/or thyroid dysfunction, not only finding a high prevalence of Vit-D deficiency in patients with AITDs, but also an inverse relationship between this deficiency and thyroid dysfunction, HT and GBD [32,33,34,35].

Explaining the link between Vit-D deficiency and AITDs is complex; in this regard, it is well-known that due to the expression of 1α-hydroxylase (CYP27B1), BL, TL, and APCs have the capacity to synthesize various Vit-D metabolites, which express certain immunomodulatory properties. In addition, it should also be noted that the expression of the VDR in these cells could suggest some type of local action of Vit-D in the immune response. The relationship between polymorphisms of the VDR or the CYP27B1 gene and the frequency of various AIDs supports these findings [32,33,34,35,36].

The influence of Vit-D on immune tolerance has been extensively studied; e.g., in APCs, Vit-D is able to modulate and reduce the secretion of pro-inflammatory cytokines (and increase that of those with an anti-inflammatory pattern), consequently inducing a shift in phenotype toward a Th2 response (instead of a Th1 or Th17 response) [36,37,38].

Vit-D also induces greater differentiation of naïve TLCD4+ into regulatory TL (Tregs), stimulating their proliferation and increasing the secretion of IL-10, TGF-β, granzymes, and perforins.

As a consequence of Vit-D deficiency, there is an increase in the secretion of TNF-α, which induces the release of CXCL10 (by thyroid cells). As a result, a positive feedback loop occurs, initiating an autoimmune response that becomes permanent over time [36,37,38].

Furthermore, Vit-D inhibits NF-κB and p38 MAPK signaling in APCs (e.g., dendritic cells), reducing the secretion of cytokines with proinflammatory activity. Vit-D also limits the proliferation and differentiation of plasma cells, the synthesis of memory globulins, and induces apoptosis of activated globulin-producing cells. The reduced number of plasma cells is associated with decreased production of immunoglobulins (IgE and IgG). In the presence of Vit-D deficiency, this process is not inhibited; consequently, immunoglobulin levels rise, leading to increased cell damage and potentially resulting in a scenario with loss of self-tolerance to thyroid antigens and hypothyroidism [38,39].

On the other hand, few studies have been conducted to assess the possible association between Mg deficiency and the risk of AITDs, suggesting that very low serum levels of Mg are associated with an increased risk of HT, hypothyroidism and positive thyroid antibodies (especially anti-Tg antibodies) [40,41].

Mg is essential for thyroid health, as it acts as a key cofactor in the conversion of T4 to active T3, thus also playing a role in maintaining an adequate TSH level. Additionally, Mg is involved in the mitochondrial oxidative phosphorylation process and in ATP synthesis, so its deficiency can lead to a decrease in iodine uptake by thyroid cells and, consequently, a decrease in the synthesis of T4 and T3 (altering the normal secretion of TSH) [40,41,42,43,44].

We documented a high prevalence of Mg deficiency (in cases), which was associated with a higher risk of developing AITDs and a higher prevalence of hypothyroidism. Additionally, we found that a cutoff point of <1.8 mg/dL (in Mg) best discriminates the risk of presenting with AITDs.

The findings of several studies suggest that Mg may play an important role in functional and/or autoimmune thyroid disorders; some findings suggest an inverse relationship with low serum Mg concentrations in individuals with positive TPOAb or TgAb (compared to those with negative TPOAbs) [40,41,42].

In other studies, the authors have shown a negative correlation between serum Mg and lymphocyte activation in patients with GBD, suggesting that immune tolerance in the thyroid may be impaired by low Mg levels [40,41,42,43].

These findings suggest that Mg may play an important role as an immune regulator, since low serum Mg levels have been associated with increased secretion of pro-inflammatory cytokines. Additionally, mutations in MAGT1 (Mg transporter 1), a specific Mg transporter, contribute to alterations in TL function and NK cell activation. Mg also promotes lymphocyte function-associated antigen 1 (LFA-1), a costimulatory molecule present on the membranes of TLCD8+ (which has antitumor properties).

It should also be noted that Mg is an enzymatic cofactor playing a fundamental role in mitochondrial oxidative phosphorylation and ATP synthesis; therefore, low levels of Mg can affect these functions and cause a decrease in thyroid iodine uptake (and, consequently, a decrease in the synthesis of thyroid hormones, causing increased TSH secretion) [40,41,42,43,44,45].

Meanwhile, authors of other studies (in patients with GBD) have found low Mg levels (compared to healthy individuals), and that this low concentration was inversely related to the activation of TLCD3+, TLCD4+, TLCD8+, and BLCD19+; this decreased immune tolerance and caused an abnormal and amplified activation of immune cells. Additionally, low Mg levels can also reduce antioxidant responsiveness and allow the accumulation of reactive oxygen species, causing oxidative stress and tissue damage [45,46,47,48,49,50].

In our study, these findings would explain (at least in part) why low serum Mg levels may be associated with a higher risk of AITDs and hypothyroidism.

On the other hand, interpreting an AUC of 0.665 along with a sensitivity of 0.928 and a specificity of 0.300 (for 25OH Vit-D levels) indicates that the model has a discriminatory capacity that has been adjusted towards a very conservative cutoff point. This describes the model as having a 66.5% probability of correctly classifying individuals with AITDs; likewise, a sensitivity of 0.928 indicates that the model is able to correctly identify 92.8% of positive cases; while a specificity of 0.300 means that 70% of healthy individuals would be incorrectly classified as positive (false positives).

In this sense, the model would have weak discrimination (although better than chance), but the chosen cutoff point (threshold) prioritizes capturing almost all positive cases (with AITDs), at the cost of including a significant proportion of false positives. Similarly, this same analysis applies to the AUC of 0.697 (sensitivity of 0.874 and specificity of 0.457) in relation to Mg levels and the risk of AITDs.

This interpretation profile is typical and frequent in medical situations where missing a positive case could have significant consequences; therefore, it is often preferable to have a test that detects the outcome of interest (even if more specific confirmatory tests are subsequently required).

Otherwise, in those participants who presented with combined Mg and 25OH Vit-D deficiency, a higher prevalence of hypothyroidism (subclinical or primary) and TPOAb and TgAb positivity was found; however, the magnitude of this association was smaller (when compared to participants with isolated Mg or 25OH Vit-D deficiency).

In this sense, it should be considered that 25OH Vit-D deficiency is more strongly associated with the presence of AITD and thyroid dysfunction.

The results of our study should be analyzed and considered in light of its potential limitations; e.g., due to the study design, we cannot establish a causal association between the deficiency of both micronutrients and the presence of thyroid dysfunction and/or AITDs; therefore, it is not possible to precisely establish a temporal sequence between the exposure (25OH Vit-D and/or Mg deficiency) and the observed outcomes.

Similarly, matching participants according to the specified potential confounding factors [age, SES, origin (urban or rural), and socioeconomic status] potentially reduces the possibility of assessing whether these variables differ between cases and controls (in addition to the potential risk of overmatching). The presence and effects of other potential residual confounding factors cannot be ruled out either, e.g., the nutritional habits of the study population in relation to the intake of foods or consumer products containing Mg and/or Vit-D, which could have potentially influenced the differences found between the study groups. The population status of other micronutrients and the presence of endocrine disruptors, which can increase (or attenuate) the associations found, were also not taken into account.

## 5. Conclusions

In conclusion, we found that Mg and Vit-D deficiency (isolated or combined) significantly increases the risk of functional thyroid disorders (specifically hypothyroidism), and AITDs. Randomized controlled trials with long-term follow-up and robust epidemiological designs are required to confirm the causal relationship between serum levels of Mg and 25OH Vit-D and other thyroid outcomes, as well as the potential effect of supplementation with these micronutrients.

## Figures and Tables

**Table 1:** Anthropometric–sociodemographic characteristics and prevalence of thyroid functioning status and positivity of thyroid autoantibodies in cases and controls.

Descriptive	Cases	Controls	Total	*p*-Value
Number of cases and controls	514	514	1028	NS
Age (years), mean ± SD	48.5 ± 17.8	47.4 ± 16.4	47.95 ± 17.1	NS
Women, prevalence (%)	63.6	63.6	63.6	NS
Low SES, prevalence (%)	65.6	65.6	65.6	NS
High SES, prevalence (%)	34.4	34.4	34.4	NS
Origin (rural/urban, prevalence (%))	38.3/61.7	38.3/61.7	38.3/61.7	NS
BMI (kg/m^2^), mean ± SD	27.5 ± 4.4	27.9 ± 4.3	27.7 ± 4.3	NS
Systolic BP (mmHg), mean ± SD	129.0 ± 12	127.4 ± 11	128.2 ± 11.5	NS
Diastolic BP (mmHg), mean ± SD	74.1 ± 7.4	72.3 ± 7.0	73.2 ± 7.2	NS

Abbreviations: BMI: body mass index; BP: blood pressure; NS: not significant; SD: standard deviation; SES: socioeconomic status.

**Table 2:** Prevalence of goiter, thyroid functional disorders, and thyroid antibody positivity (in cases and in controls).

Descriptive	Cases	Controls	*p*-Value
Number of cases and controls	514	514	NS
Prevalence (%) of goiter	64.6	11.5	<0.001
Prevalence (%) of subclinical hypothyroidism (F/M)	33.1 (35.2/29.1)	NA	<0.001
Prevalence (%) of primary hypothyroidism (F/M)	20.4 (25/17.9)	NA	<0.001
Prevalence (%) of normal thyroid function (F/M)	38.3 (40.2/38.3)	100	<0.001
Prevalence (%) of subclinical hyperthyroidism (F/M)	6.4 (7.0/4.2)	NA	<0.001
Prevalence (%) of primary hyperthyroidism (F/M)	1.8 (2.0/1.6)	NA	<0.001
Prevalence (%) of TPOAb positivity	77 (78/76)	0.0	<0.001
Prevalence (%) of TgAb positivity	50 (51/48.6)	0.0	<0.001
Prevalence (%) of TRAb positivity	20.8 (22/19.7)	0.0	<0.001

Abbreviations: F: female; M: male; Tg: thyroglobulin; TPO: thyroid peroxidase; TgAb: anti-Tg antibodies; TPOAb: anti-TPO antibodies; TRAb: anti-thyrotropin receptor antibodies; NS: not significant; NA: not applicable.

**Table 3:** 25OH Vit-D and Mg levels and prevalence of deficiency (of both) in cases and controls (and according to age).

Descriptive	Cases	Controls	Total	*p*-Value
Number of cases and controls	514	514	1028	NS
25OH Vit-D, mean ± SD	25.1 ± 9.0	31.3 ± 7.7	28.2 ± 8.9	<0.001
25OH Vit-D, mean ± SD (age < 50 years)	25.9 ± 9.5	32.3 ± 7.7	29.1 ± 9.2	<0.001
25OH Vit-D, mean ± SD (age ≥ 50 years)	24 ± 8.2	30 ± 7.6	27 ± 8.4	<0.001
Prevalence (%) of 25OH Vit-D deficiency (value < 20 ng/mL)	37.5	6.4	22	<0.001
Prevalence (%) of 25OH Vit-D deficiency (value < 12 ng/mL)	5.1	0.6	2.8	<0.001
Prevalence (%) of 25OH Vit-D deficiency (age < 50 years)	36.5	5.3	20.9	<0.001
Prevalence (%) of 25OH Vit-D deficiency (age ≥ 50 years)	38.9	7.9	23.4	<0.001
Magnesium, mean ± SD	1.9 ± 0.2	2.1 ± 0.2	2.0 ± 0.2	<0.001
Magnesium, mean ± SD (age < 50 years)	1.91 ± 0.23	2.05 ± 0.16	1.99 ± 0.21	<0.001
Magnesium, mean ± SD (age ≥ 50 years)	1.89 ± 0.27	2.0 ± 0.2	1.96 ± 0.25	<0.001
Prevalence (%) of Mg deficiency (value < 1.7 mg/dL)	21.1	5.1	13.1	<0.001
Prevalence (%) of Mg deficiency (age < 50 years)	17.2	3.2	10.2	<0.001
Prevalence (%) of Mg deficiency (age ≥ 50 years)	25.9	7.5	16.7	<0.001
Combined prevalence (%) of Mg deficiency (<1.7 mg/dL) and 25OH Vit-D deficiency (<20 ng/dL)	18.5	1.4	9.9	<0.001

Abbreviations: 25OH Vit-D: 25-hydroxyvitamin D; Mg: magnesium; SD: standard deviation; NS: not significant.

**Table 4:** Prevalence of thyroid Abs, in relation to 25OH Vit-D and Mg levels.

25OH Vit-D and/or Mg Levels	Prevalence (%) of Negative (−) or Positive (+) Thyroid Antibodies					
25OH Vit-D levels	% TPOAb (−)	% TPOAb (+)	% TgAb (−)	% TgAb (+)	% TRAb (−)	% TRAb (+)
20–50 ng/mL	68.5	31.5	80.4	19.6	92.3	7.7
≥12 to <20 ng/mL	36.3	63.7 *	55.3	44.7	80.1	19.9
<12 ng/mL	31	69 **	62.1	37.9	75.9	24.1
Mg levels	TPOAb (−)	TPOAb (+)	TgAb (−)	TgAb (+)	TRAb (−)	TRAb (+)
1.7–2.4 mg/dL	64.9	35.1	76.6	23.4	90.6	9.4
<1.7 mg/dL	38.1	61.9 †	64.2	35.8	82.8	17.2
Mg levels < 1.7 mg/dL and 25OH Vit-D < 20 ng/mL)	33.4	64.6 ††	41.7	58.3 †††	79.1	20.1

* OR: 3.8 (CI: 95%: 2.8–5.2; *p* < 0.001); ** OR: 4.1 (CI: 95%: 2.7–5.0; *p* < 0.001); † OR: 3.0 (95% CI: 2.1–4.4; *p* < 0.001); †† OR: 3.1 (95% CI: 1.8–4.4; *p* < 0.001); ††† OR: 2.3 (95% CI: 1.4–3.5; *p* < 0.001). Abbreviations: 25OH Vit-D: 25-hydroxyvitamin D; Mg: magnesium; Tg: thyroglobulin; TPO: thyroid peroxidase; TgAb: anti-Tg antibodies; TPOAb: anti-TPO antibodies; TRAb: anti-thyrotropin receptor antibodies.

## Data Availability

The original contributions presented in this study are included in the article. Further inquiries can be directed to the corresponding author.
